# Corrigendum: Phosphate steering by Flap Endonuclease 1 promotes 5′-flap specificity and incision to prevent genome instability

**DOI:** 10.1038/ncomms16145

**Published:** 2017-08-07

**Authors:** Susan E. Tsutakawa, Mark J. Thompson, Andrew S. Arvai, Alexander J. Neil, Steven J. Shaw, Sana I. Algasaier, Jane C. Kim, L. David Finger, Emma Jardine, Victoria J. B. Gotham, Altaf H. Sarker, Mai Z. Her, Fahad Rashid, Samir M. Hamdan, Sergei M. Mirkin, Jane A. Grasby, John A. Tainer

Nature Communications 8 Article number: 15855 ; DOI: 10.1038/ncomms15855 (2017); Published: 06 27 2017; Updated: 08 07 2017

The financial support for this Article was not fully acknowledged. The Acknowledgements should have included the following:

This research used resources of the Advanced Light Source and the Stanford Synchrotron Radiation Lightsource, which are DOE Office of Science User Facilities under contract no. DE-AC02-05CH11231 and DE-AC02-76SF00515, respectively. The SSRL Structural Molecular Biology Program is supported by the DOE BER, and by the NIH (including P41GM103393). The SIBYLS beamline 12.3.1 is supported by the IDAT program from the DOE BER and the NIH project MINOS (R01GM105404).

