# Supplementary material for: Phosphate steering by Flap Endonuclease 1 promotes 5′-flap specificity and incision to prevent genome instability
Source: Nat Commun. 2017 Jun 27;8:15855. doi: 10.1038/ncomms15855 (PMC5490271; doi:10.1038/ncomms15855)
Supplement: Supplementary Information — Supplementary Figures, Supplementary Table and Supplementary References [file ncomms15855-s1.pdf]

Supplementary Figure 1 *Related to Figures 1,3,4.*

A

Code	Sequence
T	5'-GTG TCG AGC AGT CCT TGT GAC GAC GAA GTC GTC C-3'
F1	5'-FAM-TT TTT ACA AGG ACT GCT CGA CAC-3'
E1	5'-Phos-ACA AGG ACT GCT CGA CAC-FAM-3'
E2	5'-ACA AGG ACT GCT CGA CAC-FAM-3'
E3	5'-FAM-ACA AGG ACT GCT CGA CAC-3'
CT	5'-ACT CTG CCT CAA GAC GGT-3'
CF1	5'-ACC GTC C-3'
CF2	5'-TTA ATT GAG GCA GAG T-3'
CF3	5'-AAC TTT GAG GCA GAG T-3'
CF4	5'-A TAT CTT GAG GCA GAG T-3'

B

Substrate	Composition
S5,1	F1 + T
S0,1-5P	E1 + T
S0,1-5OH	E2 + T
S0,1-5FAM	E3 + T
S4,1-R100A	CF1 + CF2 + CT
S4,1-D233N	CF1 + CF3 + CT
S5,1-D86N	CF1 + CF4 + CT

C

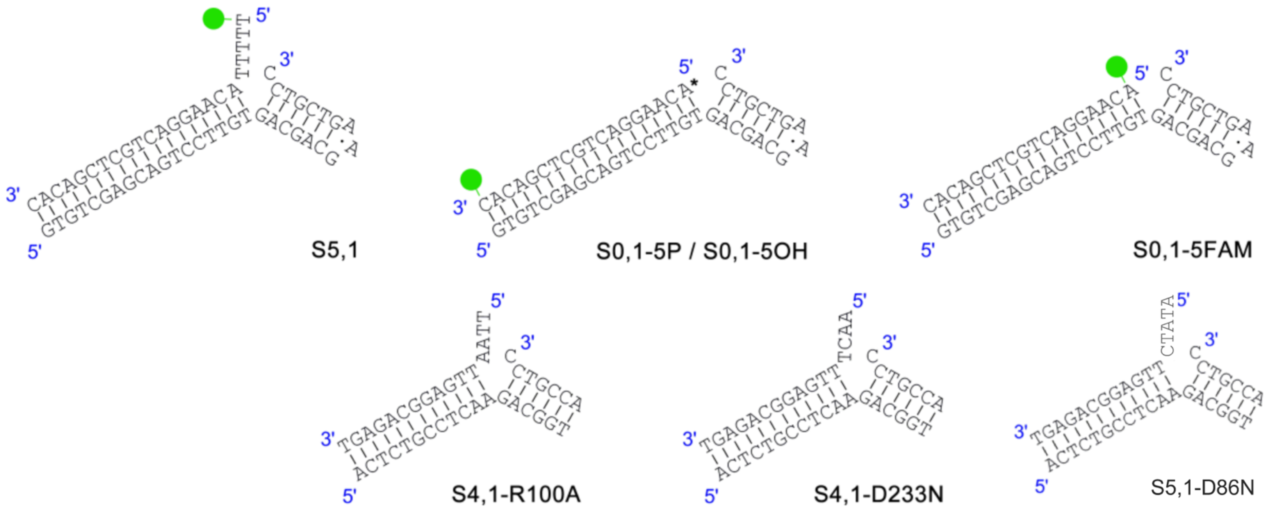

Supplementary Figure 1 DNA substrates for the kinetics assays and crystallography (A) Sequences of individual oligonucleotides used (FAM = terminal 6-carboxyfluorescein; Phos = phosphate). (B) Combinations of oligonucleotides annealed to prepare the various substrate constructs. (C) Structures of the substrates; green circle represents position of FAM label in kinetics substrates (top row). Asterisk (*) in middle structure corresponds to 5′-phosphate (for S0,1-5P) or 5′-OH (for S0,1-5OH), respectively. S5,1-D86N, S4,1-R100A and S4,1-D233N (bottom row) are the substrates co-crystallized with the R100A, D233N, and D86N mutants of FEN1, respectively.

Supplementary Figure 2 *Related to Figures 1 and 3.*

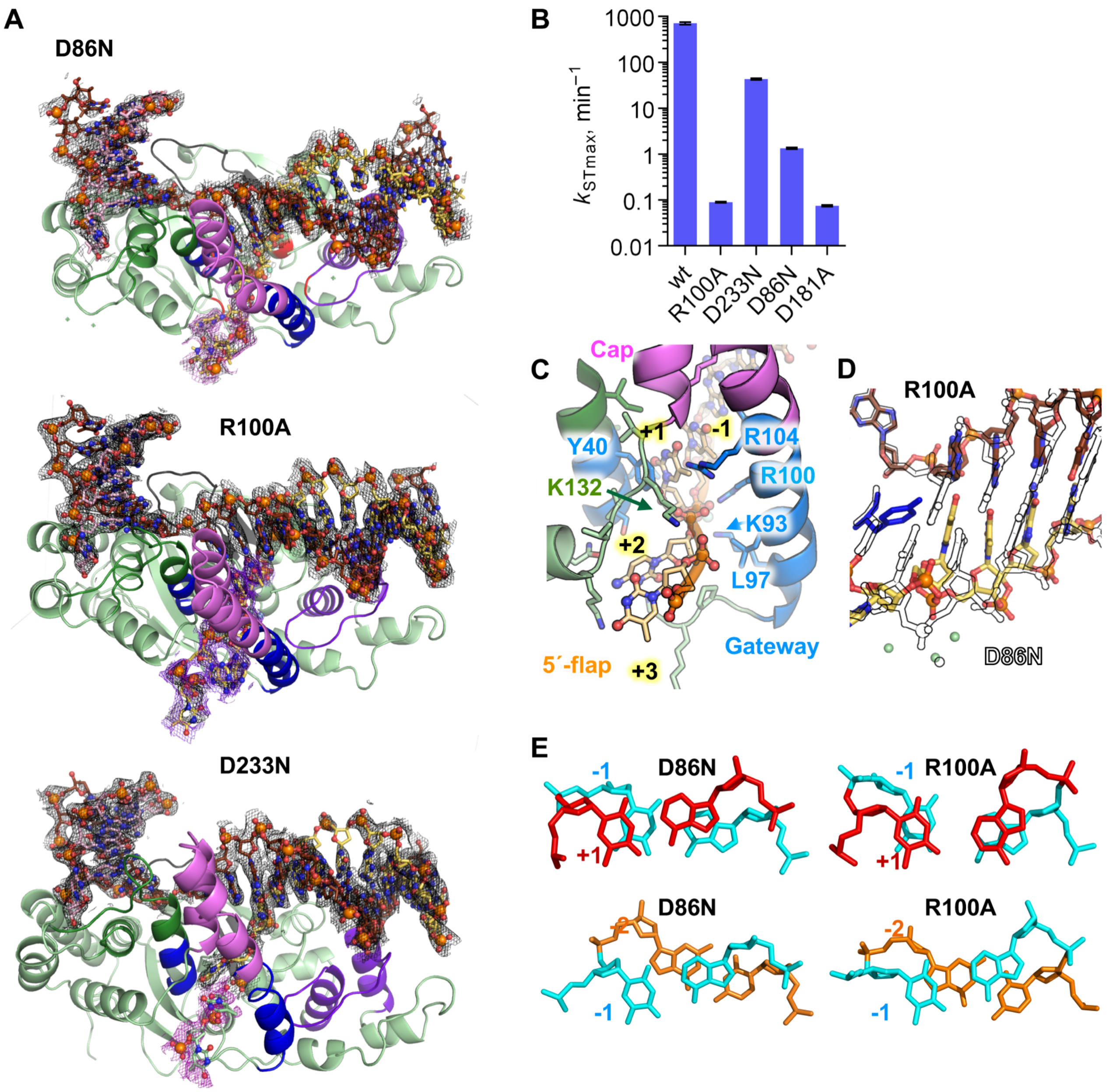

Supplementary Figure 2 Structural and Biochemical Analysis of hFEN1 mutants (A) Simulated annealing 2mFo-DFc omit maps of DNA overlaid on hFEN1-D86N, hFEN1-R100A, and hFEN1-D233N structures. Maps for each chain are shown in black at 0.5 sigma, 2.0 Å carving. Maps for the ssDNA 5′-flap region only (R100A structure) or the cleaved 5′-flap fragment (D233N structure) are shown in purple at 0.2 sigma, 2.0 Å carving. (B) Single turnover analysis of hFEN1 active site mutants. Incision assays were done twice in duplicate but with independent enzyme dilutions for each tube. Error bars are shown as a function of standard error of the mean (SEM), with 4 replicates. (C) The van der Waals and electrostatic interactions to the ss 5' flap is non-sequence specific. (D) Overlay of hFEN1-D86N (outline) onto hFEN1-R100A (colored by element) to show the shift of the DNA onto the metal and the rotation of the Tyr40. (E) In the hFEN1-D86N structure, the DNA near the scissile phosphate is distorted, leading to lower intrastrand base stacking and increased interstrand stacking. The DNA from R100A is shown for comparison.

Supplementary Figure 3 *Related to Figures 1-3.*

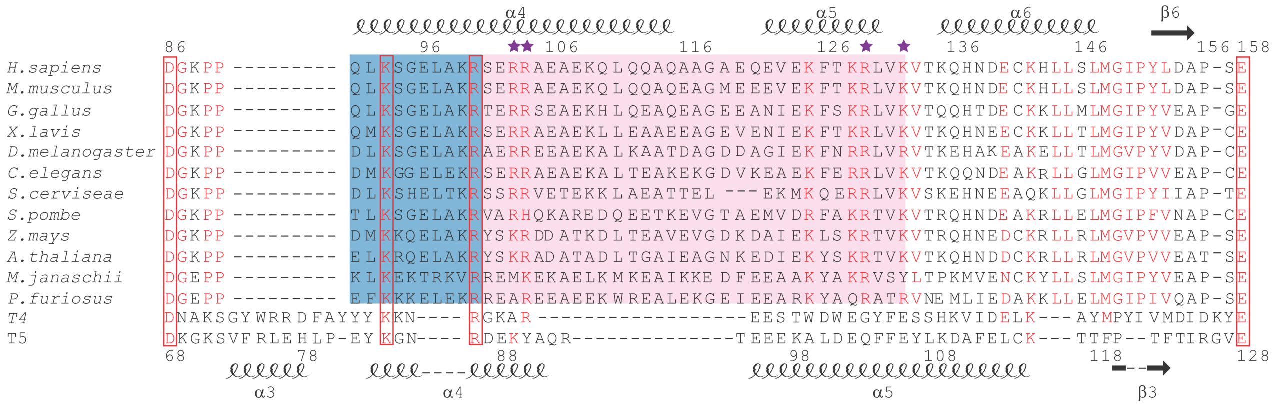

Supplementary Figure 3 Structure-based sequence alignment of FEN1 family Shown is a subset of sequence from and alignment of 191 non-redundant FEN1 sequences from phage, eubacterial, archaeal and eukaryotic FENs. The sequences span from the first and last conserved carboxylate residues before and after the helical arch, respectively. Numbering on the top and bottom is relative to human FEN1 and T5 phage 5′ nuclease, respectively. Helix and β-sheet secondary structural elements are shown as coils and arrows, respectively, and those on the top and bottom are based on the human FEN1 (3Q8L) and T5 5′ nuclease (5HML) structures in the presence of DNA, respectively. Invariant residues from an alignment of 191 non-redundant FEN1 sequences are shown in red with a red box around them. These include the two active site carboxylates that are on either side of the helical arch (human Asp86, Glu158) and two active site basic residues (human Lys93, Arg100). Other residues that show a high percentage of semiconservation (i.e., Lys or Arg) from archaea to humans are shown in red as well. Those that have been mutated in human for this study are highlighted with a purple star. With respect to human FEN1 numbering, Arg104 is conserved in 94% of the sequences, and those that do not show an Arg104 alignment have a Lys or Arg residue that aligns to Arg103. Arg103 is 71% conserved from archaea to humans, with those lacking an Arg103 cognate being sequences from archaea and protozoa (i.e., more conserved in the higher eukarya – plants, fungus and animals). Arg129 and Lys132 show 85% and 86% conservation, respectively, among the sequences that were aligned, and those not displaying conservation being clustered within archaea and protozoa with exceptions from a fungus and zebrafish. Note the general lack of conservation of these four basic residues in the phage 5′ nucleases (T5 and T4), which exhibit significant secondary structural differences in this region and are known to have inadvertent cleavage in the 5′-flaps of certain substrates.

Supplementary Figure 4 *Related to Figures 4 and Supplementary Figure 1 (caption on next page).*

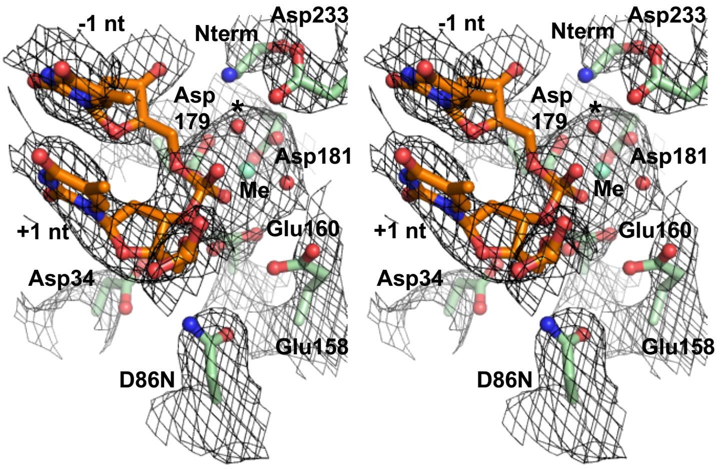

*	Attacking Water
Me	Metal

Supplementary Figure 4 Stereoview of Active Site Residues in hFEN1-D86N Stereoview shows how a water molecule is positioned for linear attack on the scissile phosphate. The simulated annealing 2mFo-DFc omit map is drawn at 0.7 sigma, 2.0 Å carving. The two waters in the active site were placed based on positive density in the mFo-DFc map at the last stage of the refinement.

Supplementary Figure 5. *Related to Figures 4 and Supplementary Figure 1 (caption on next page).*

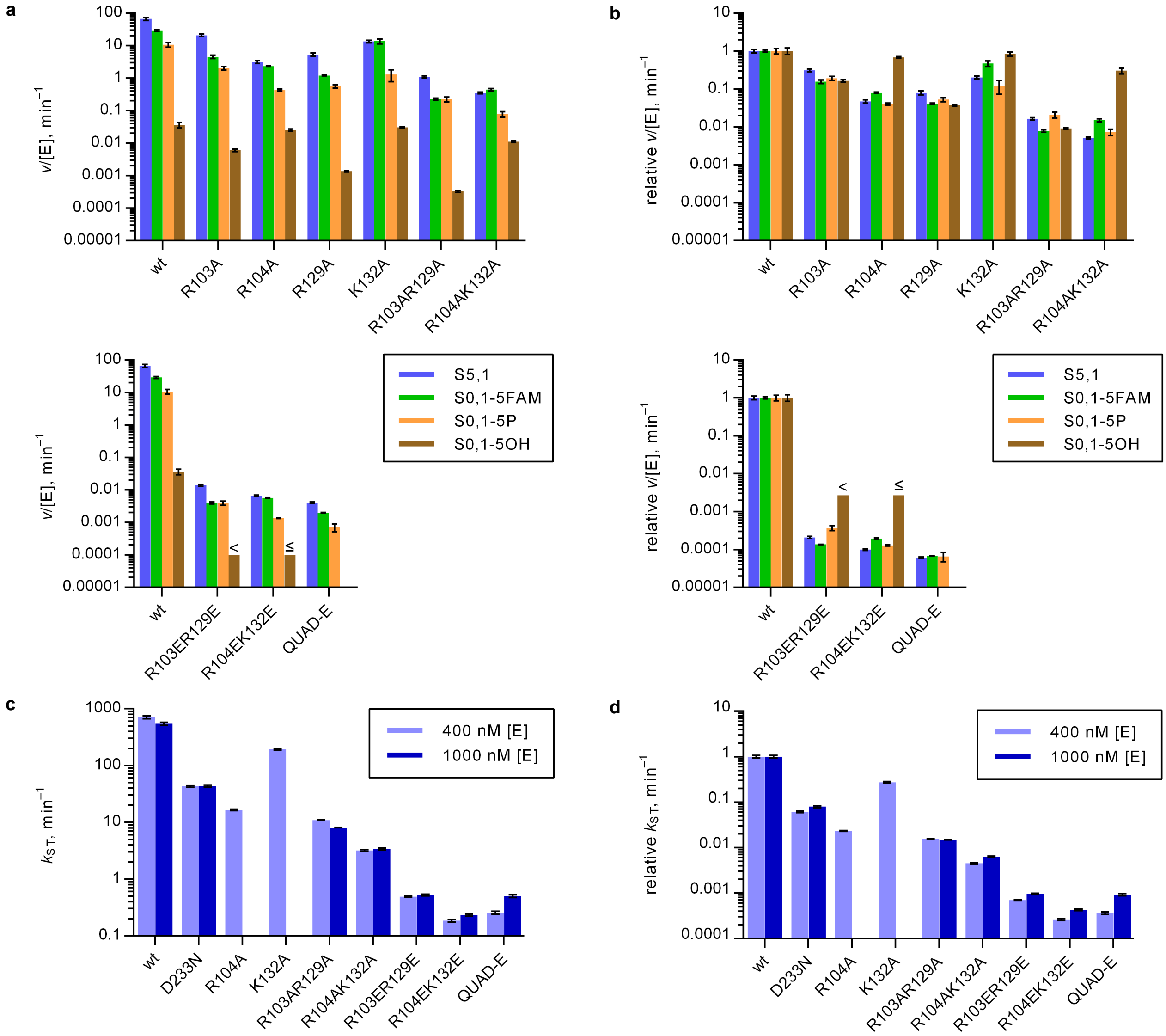

e

Protein	*v*/[E] S5,1	*v*/[E] S0,1-5P	*v*/[E] S0,1-5OH	*v*/[E] S0,1-5FAM	k_{ST} 400 nM [E]	k_{ST} 1,000 nM [E]
wt hFEN1	5	3	6	5	N = 2, n = 6	N = 2, n = 6
R103A	4	3	3	4	—	—
R104A	4	3	3	6	N = 2, n = 6	—
R129A	4	3	3	4	—	—
K132A	4	3	3	3	N = 2, n = 6	—
R104AK132A	5	3	3	4	N = n = 5	N = n = 5
R103AR129A	3	3	3	4	N = 2, n = 6	N = 1, n = 3
R103ER129E	4	3	—	4	N = n = 3	N = n = 6
R104EK132E	4	3	—	4	N = n = 3	N = n = 6
QUAD-E	4	3	—	4	N = n = 6	N = n = 8
D233N	—	—	—	—	N = 2, n = 6	N = 1, n = 3

Supplementary Figure 5. FEN1 kinetics experiments with four different FEN1 substrates. (A) Normalised rates for cleavage of indicated substrates (at 50 nM) under multiple turnover conditions, by wt FEN1 and mutants shown (A-mutants in upper graph; E-mutants in lower graph). Rates were measured by either dHPLC or capillary electrophoretic analysis of quenched reaction samples, as outlined in Online Methods. (B) The same results as in panel a, but expressed relative to the reaction rate for wt FEN1 with each substrate. (C) Measured reaction rates for hydrolysis of substrate S5,1 (at 5 nM) by either 400 nM or 1,000 nM of each enzyme indicated, under single turnover conditions as described in the Online Methods section. Values were derived by regression in GraphPad Prism 6.05 using either a one-phase or two-phase association model, with model selection determined by statistical analysis using Aikake's Information Criteria (AIC). (D) The same data as in panel c shown relative to the rate for wt FEN1 at each enzyme concentration. (E) Replicate information for experiments presented in panels *a*–d. For multiple turnover measurements (i.e. *v*/[E] values), and single turnover measurements carried out using manual sampling, individual biological replicates (N) were performed. For single turnover measurements using rapid quench flow apparatus, technical triplicates (n) were carried out once (N = 1) or twice (N = 2).

Supplementary Figure 6. *Related to Figure 4.*

a

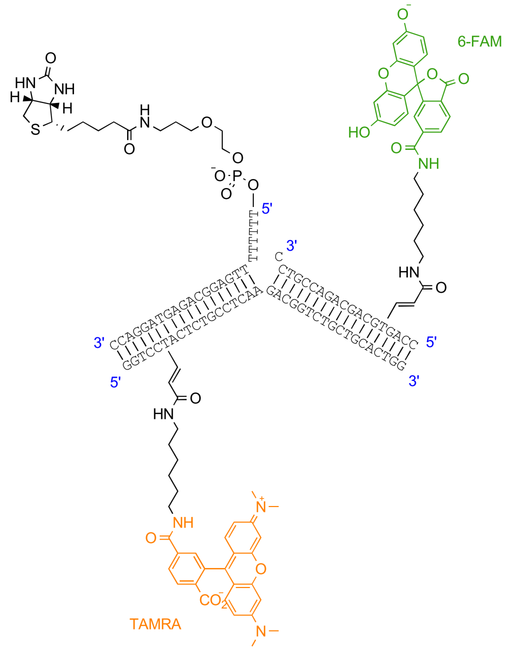

b

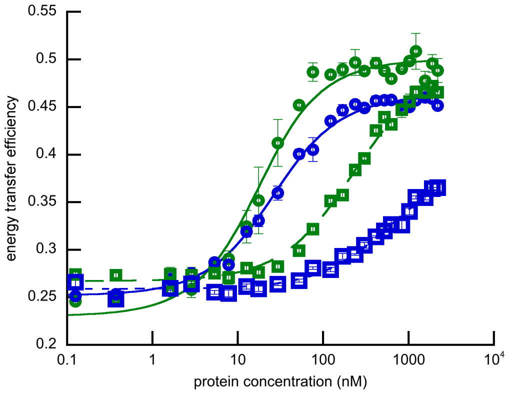

c

Protein	K_d, nM S6,1-FRET	K_d, nM S0,1-5P-FRET	K_d, nM S0,1-5OH-FRET
wt hFEN1	*12.6 ± 1.7*	11.9 ± 1.1	256 ± 13
R104AK132A	44.9 ± 3.6	*21.4 ± 1.2*	—
R103AR129A	19.5 ± 1.2	19.3 ± 1.0	—
R103ER129E	45.5 ± 2.3	—	—
R104EK132E	59.3 ± 3.1	—	—
QUAD-E	*227 ± 16*	—	—

d

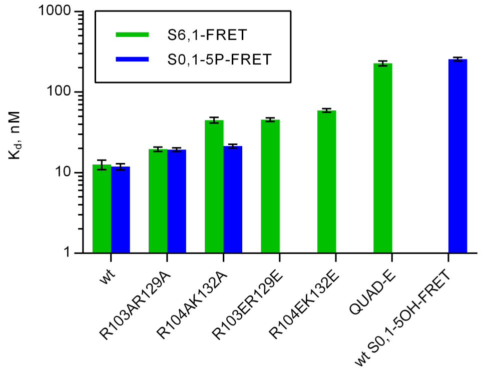

Supplementary Figure 6. Details of FRET binding studies, performed as described[1]. (A) Structure of the double-flap substrate S6,1-FRET, showing the position of labels and linker chemistry used. The single flap FRET substrates S0,1-5P-FRET and S0,1-5OH-FRET lack the 5′-terminal biotin and TTTTTT sequence, replacing it with a 5′-phosphate or 5′-OH group, respectively, in an analogous manner to the kinetics substrates. Note that the substrates S6,1-FRET; S0,1-5P-FRET; S0,1-5OH-FRET correspond exactly to the constructs named DF(DAL); SF(DAL); HO-SF(DAL), respectively, in [1]. (B) Example binding curves obtained by titration of enzyme into 10 nM S6,1-FRET, with with wt FEN1 (open circles) or the QUAD-E mutant (open squares), in either Ca^{2+}-containing buffer (green) or EDTA-containing buffer (blue). (C) Equilibrium dissociation constants, $K_{d(bending)}$, derived for binding of either the double- or single-flap FRET substrates to each protein, as indicated. Experiments were each performed once as technical triplicate measurements, except for values in italic, which are derived from global fitting of data from two such triplicate experiments; in either case, errors given are standard errors derived during regression analysis. (D) Graphical representation of the binding data tabulated above.

Supplementary Figure 7. *Related to Figure 5.*

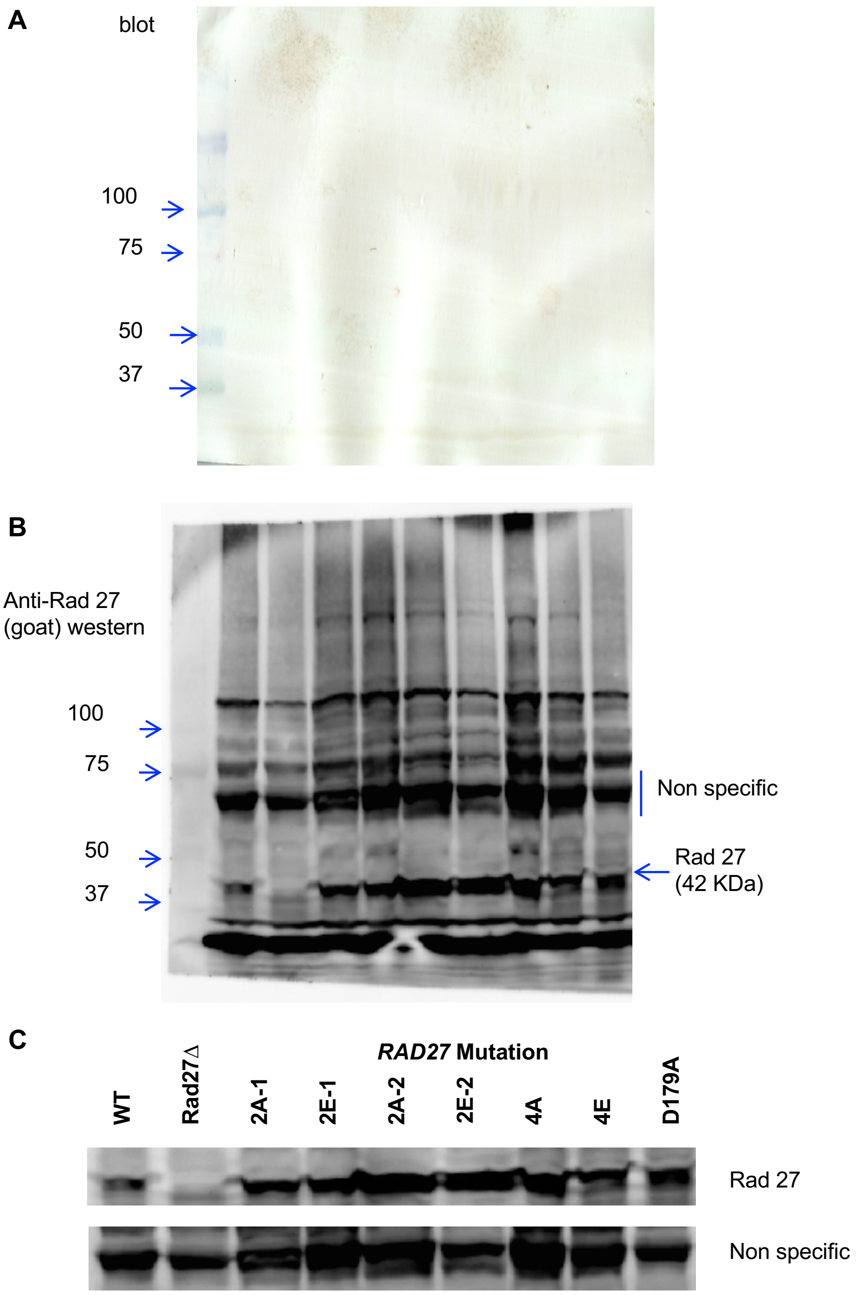

Supplementary Figure 7 Western Blot of Rad27 WT and mutant proteins Rad27 protein expression was not substantially altered in the mutated strains. (A) Picture of the membrane showing marker positions. (B) Uncropped Western blot with anti-Rad27 antibody. (C) Rad27 stained band and non-specific protein used in analysis. *Related to Figure 5.*

Supplementary Table 1

Genotype information for wt and mutant *Rad27* strains. *Related to Figure 5.*

Strain name	Equivalent human FEN1 mutation(s)	Yeast genotype
YJK212	wt	*MATa leu2-Δ1 trp1-Δ63 ura3–52 his3–200 bar1::HIS3 III(75594–75641)::URA3-Int-(GAA)$_{100}$-TRP1 XI(224681–224712)::hphMX4*
**Following strains have the same background genotype as YJK212 with specific rad27 alleles*		
YJK214	D181A	*Rad27-D179A*
YJK215	R104A K132A	*Rad27-R105A K130A*
YJK217	R104E K132E	*Rad27-R105E K130E*
YJK227	R103A R129A	*Rad27-R104A R127A*
YJK230	R103E R129E	*Rad27-R104E R127E*
YJK220	R103A R104A R129A K132A	*Rad27-R104A R105A R127A K130A*
YJK218	R103E R104E R129E K132E	*Rad27-R104E R105E R127E K130E*

SUPPLEMENTARY REFERENCES

1. Algasaier SI*, et al.* DNA and protein requirements for substrate conformational changes necessary for human flap endonuclease-1-catalyzed reaction. *J Biol Chem* **291**, 8258-8268 (2016).
